# A Nationwide Study of Inpatient Case Rate Incidence of Cannabis-Related Diagnoses in Switzerland

**DOI:** 10.3389/ijph.2022.1605554

**Published:** 2022-12-21

**Authors:** Philippe Pfeifer, Reto Auer, Stéphanie Baggio, Franz Moggi

**Affiliations:** ^1^ University Hospital of Psychiatry and Psychotherapy, University of Bern, Bern, Switzerland; ^2^ Institute of Primary Health Care (BIHAM), University of Bern, Bern, Switzerland; ^3^ Centre for Primary Care and Public Health (Unisanté), Lausanne, Switzerland; ^4^ Division of Prison Health, Geneva University Hospital and University of Geneva, Geneva, Switzerland

**Keywords:** mental health, prevention, prevalence, cannabis-related diagnoses, hospitalization rates

## Abstract

**Objectives:** Cannabis is the most commonly used illicit substance and has been associated with mental health issues. In this study, we examined trends in hospitalizations due to cannabis use.

**Methods:** Data were obtained from the Swiss Federal Statistics Office and comprised hospital main diagnoses, gender, age group and region of all psychiatric inpatient cases in Switzerland from 1998 to 2020. We performed trend analyses of annual case rates with cannabinoid-related diagnoses and compared them to trend analyses of alcohol-related and psychotic disorders.

**Results:** Case rates of CRDs significantly increased in the observed time period. From all psychiatric main diagnoses, CRDs were overrepresented in the age groups of 15–24 and 25–44 years.

**Conclusion:** We found a sharp increase in hospitalizations for CRD. Future studies should test whether changes in the upcoming cannabis regulation, which can facilitate the implementation of interventions to address mental health among users, impact future hospitalization rates of CRD.

## Introduction

Tetrahydrocannabinoid (THC), the main active component in cannabis plant products, is the most commonly used illicit psychoactive substance worldwide [1–3]. Over the past decade, the regulatory framework for purchasing cannabis has rapidly changed and, following the trends in Uruguay, Canada, and the United States, an increasing number of Western societies have discussed or taken action to regulate cannabis in their local markets [4, 5]. In Switzerland, cannabis was decriminalized in 2013 but has not officially been regulated. Overall, the impact of legalization on the frequency and quantity of cannabis use is controversial and no robust recommendations are currently available [6]. In the Swiss population aged 15–64 years, there was a slight increase in monthly cannabis use between 1992 (2.9%) and 2017 (4%). Young people (aged 20–24 years) and men had the highest prevalence rate of cannabis use [7]. The Swiss Monitoring System of Addiction and Non-communicable Diseases reported an overall increase in hospital stays with a primary or secondary diagnosis of cannabis-related diagnoses (CRD) between 2012 and 2020 [8]. However, detailed information on whether the hospitalization trends were due to CRD as a primary mental health-related disorder or a comorbid condition is lacking.

Concerns about the detrimental mental health effects of cannabis use have been expressed worldwide since its first appearance in the illegal drug market [9, 10]. The latest World Drug Report of the United Nations reported on the negative impacts of cannabis use on public health, including an increase in hospitalization, the intensity of cannabis use (frequency and quantity), and THC content in cannabis products [11]. Regarding mental healthcare, severe conditions attributed to cannabis use may also require inpatient psychiatric treatment. Recently, two studies from Canada and Germany showed an increase in psychiatric hospitalizations in the last 10 years because of CRD, particularly for cannabis dependence and cannabis-associated psychosis [12, 13].

To assess whether similar trends have occurred in Switzerland, we analyzed a nationwide database of inpatient diagnoses and their frequencies annually from 1998 to 2020 in relation to the general population in Switzerland. Alcohol-related disorders and schizophrenia spectrum disorders were used as comparison conditions, as they represent other important substance-related psychiatric diagnoses in Switzerland. Furthermore, we have included additional comparisons based on sex and age group.

## Methods

### Ethical Approval

Anonymized data was used for this study; therefore, no approval from the local ethics committee was required.

### Data Collection

For the present longitudinal observational study, data were obtained from the Swiss Federal Statistical Office (FSO). The FSO is an authority of Switzerland and part of the Federal Department of Home Affairs (https://www.bfs.admin.ch/bfs/de/home.html). The FSO is Switzerland’s national competence center for official statistics. It produces and publishes statistical information on the status and development of the population, economy, society, education, research, territory, and environment. Information from the FSO is used for opinion building among the population and for planning and managing key policy areas. Data from the FSO are available to researchers and institutions for an administrative charge. Data on the development of the Swiss population was extracted from the FSO data bank, which is accessible to the public [14].

### Data

We ordered data from the FSO on the absolute annual frequencies of all psychiatric diagnoses and diagnosis-related inpatient cases treated in Switzerland between 1998 and 2020. Data regarding outpatient therapy and rehabilitation were not included. The FSO data is managed in such a way that every new admission to a clinic that results in inpatient treatment (treatment > 24 h) generates a new case. Therefore, in the present study, a “case” is defined as every new admission resulting in inpatient treatment in Switzerland between 1998 and 2020. Aggregated case data assigned to a specific individual were not analyzed in this study.

We used the primary diagnosis that was associated with treatment at a clinic, defined according to the International Statistical Classification of Diseases and Related Health Problems, Tenth Revision (ICD-10) [15]. CRD included all ICD-10 diagnostic codes F12.0-12.9. We included the following specific CRDs: acute intoxication, harmful use, dependence syndrome, withdrawal state, psychotic disorder, and residual diagnosis (amnesic syndrome, residual and late-onset psychotic disorder, other mental and behavioral disorders, and unspecified mental and behavioral disorders). The degree of cannabis consumption was assessed clinically during the hospital stay. For alcohol-related diagnoses, the ICD-10 diagnostic code F10 was used, while for psychosis disorders, ICD-10 diagnostic codes F20–29 (schizophrenia-spectrum disorders) were used.

### Data Analysis

Case rates were calculated as the annual number of CRD cases in proportion to the annual general population in Switzerland between 1998 and 2020.

Trend analyses were performed on all annual case rates of CRD from 1998 to 2020 (23 years). Separate trend analyses were also performed for subgroups according to sex, age group, and language region; however, as the proportions did not significantly change within the Swiss population from 1998 to 2020, we did not calculate the specific case rates for these subgroups [14]. Stata 17 was used for the regression analyses to assess linear and quadratic trends for case frequencies of mental and behavioral disorders resulting from cannabinoid disorders. Bootstrapped standard errors were used to account for data clustering. To obtain a smoothed-curve representation of the data, we used a Hadrick-Prescott Filter. Slope coefficients were considered significant at *p* < 0.05.

## Results

**FIGURE 1:**
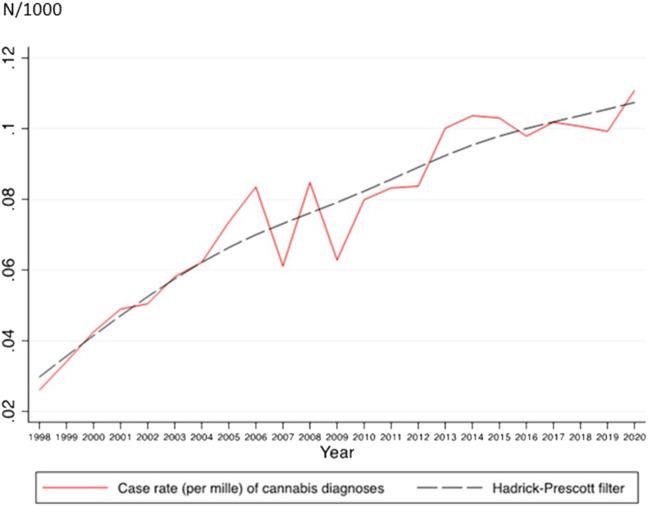
Annual case rates and estimated quadratic trend line for inpatient cases with cannabis-related diagnosis (A Nationwide Study of Inpatient Case Rate Incidence of Cannabis-Related Diagnoses in Switzerland, Switzerland, 2022).

From 1998 to 2020, 2.15 million inpatient cases with psychiatric diagnoses were found, of which 13,984 were hospitalized with CRD, 332,743 with an alcohol-related diagnosis, and 283,254 with a psychosis-related diagnosis. A mean of 627.2 cases (standard error [SE]: 223.9) of CRDs was found. The case rates of CRDs (F12.0–12.9) increased during the observation period (Figure 1). For further details, see Supplementary Tables S1, S2.

The case rate of any type of CRD significantly increased linearly over time between 1998 and 2020 (Table 1). The effect size was very high (*R* ^2^ = 914%). A significant negative quadratic trend was also seen, suggesting that the increase in the case rates of CRD has slowed down in recent years.

**TABLE 1:** Trend analyses for case rates of cannabis related diagnoses and comparators (A Nationwide Study of Inpatient Case Rate Incidence of Cannabis-Related Diagnoses in Switzerland, Switzerland, 2022).

Main psychiatric diagnosis (ICD-10)		Linear trend		Quadratic trend		Adj.R-square
		*B*	*p*	*b*	*p*	
Mental and behavioral disorders due to cannabinoid use (F12.0-9)	All types (F12.0-9)	0.0035	<0.001	−0.0011	0.009	0.91
	Acute intoxication (F12.0)	0.953	<0.0001	0.507	<0.0001	0.06
	Harmful use (F12.1)	0.0003	<0.001	−0.0001	0.002	0.67
	Dependence syndrome (F12.2)	0.0024	<0.001	−0.0008	0.001	0.91
	Withdrawal state (F12.3)	0.0001	<0.001	0.160	<0.0001	0.56
	Psychotic disorder (F12.5)	0.042	<0.0001	0.646	<0.0001	0.07
	Residual categories (F12.X)*	0.0006	<0.001	0.849	<0.0001	0.61
Mental and behavioral disorders due to alcohol use (F10.0-9)	0.0206	<0.001	−0.0039	<0.001	0.68	
Schizophrenia, schizotypal and delusional disorders (F20-29)	0.0015	0.793	−0.0032	0.011	0.28	

*F12.X includes: F12.6 (amnesic syndrome), F12.7 (residual and late-onset psychotic disorder), F12.8 (other mental and behavioral disorders ) and F12.9 (unspecified mental and behavioral disorders).

The case rates of inpatients with harmful cannabinoid use, cannabinoid dependence, withdrawal states, and psychotic disorders due to cannabinoid use statistically significantly increased between 1998 and 2020. Quadratic trends were significant for harmful use and dependence on cannabis. The negative estimates suggested that the growth slowed over time (Supplementary Figure S1). The case rates of alcohol-related diagnoses had a significant positive linear trend and a negative quadratic trend, suggesting an increase over time that also slowed over time. While no significant linear trend in the case rates for schizophrenia spectrum disorders was found (Table 1), a negative quadratic trend was seen.

**FIGURE 2:**
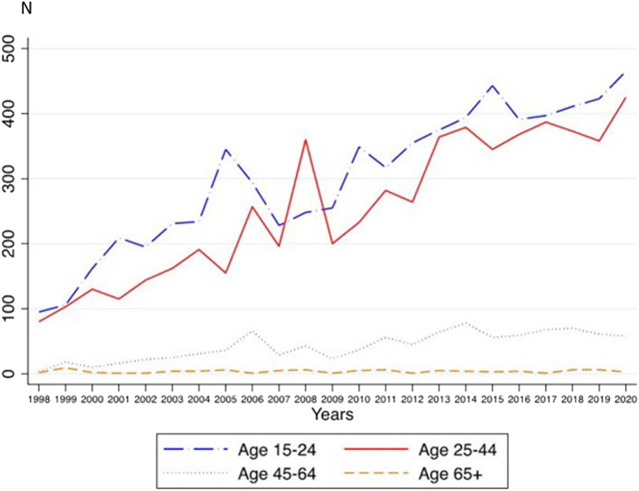
Annual frequencies for inpatient cases with cannabis-related diagnoses in different age groups (A Nationwide Study of Inpatient Case Rate Incidence of Cannabis-Related Diagnoses in Switzerland, Switzerland, 2022).

Among all the CRD cases, 91.5% were included in the 15–24 (49%) and 25–44 (42.5%) age groups. Case frequencies within the 15–24 age group increased from 7.9% to 17.1% (mean: 12.1, 95% CI: 10.3–13.9). In the 25–44 age group, cases increased from 1.1% to 3.6% (mean: 2.2, 95% CI: 1.8–2.6) (Supplementary Table S1). We also performed trend analyses for age-related CRDs. Trend analyses showed a statistically significant linear increase in the frequency of cases in the 15–24 age group (*b* = 14.83, *p* < 0.001, adj.R-square = 0.88), 25–44 age group (*b* = 15.04, *p* < 0.001, adj.R-square = 0.86), and 45–64 age group (*b* = 2.72, *p* < 0.001, adj.R-square = 0.73), but not for the 65+ age group (*b* = 0.03, *p* = 0.726) (Figure 2). Quadratic trends were not significant (age 15–24: *b* = −0.28, *p* = 0.245; age 25–44: *b* = −0.15, *p* = 0.514; age 45–64: *b* = −0.09, *p* = 0.212, and age 65+: *b* < 0.01, *p* = 0.926). The increase was similar in the 15–24 and 25–44 age groups (overlapping CIs for the linear trends: age 15–24: 13.34; 16.32, age 25–44: 13.57; 16.50) but lower in the 45–64 age group (2.15; 3.30).

**FIGURE 3:**
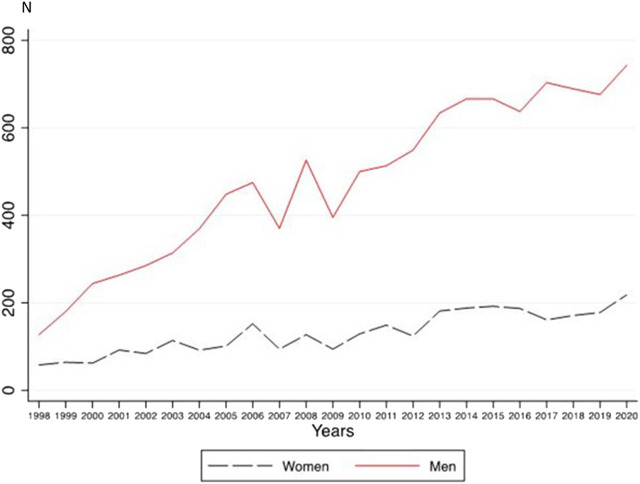
Annual frequencies for inpatient cases with cannabis-related diagnoses in women and men (A Nationwide Study of Inpatient Case Rate Incidence of Cannabis-Related Diagnoses in Switzerland, Switzerland, 2022).

In 1998, the women-to-men quotient of all cases with CRD was .46, while in 2020, it was .29 (Supplementary Table S1). For women, a linear trend was found (*b* = 6.37, *p* < 0.001, adj.R-square = .81), but no quadratic trend (*b* = −0.01, *p* = 0.923). For men, there were both linear and quadratic trends (linear: *b* = 26.23, *p* < 0.001; quadratic: *b* = −0.48, *p* = 0.040, adj.R-square = 0.94) were found. The linear increase was significantly greater in men compared to women (95% CIs for the linear trend: 5.24; 7.51 for women and 24.20; 28.27 for men) (Figure 3).

We also compared case frequencies in the German language region to those in the Latin language region (French and Italian speaking cantons) of Switzerland and found no statistically significant differences (a quotient of 1.5 in 1998 compared to a quotient of 1.4 in 2020). Both regions had linear (Latin: *b* = 12.64, *p* < 0.001, adj.R-square = 0.80; German: 19.96, *p* < 0.001, adj.R-square = 0.96) and quadratic (Latin: −0.97, *p* < 0.001; German: *b* = 0.48, *p* = 0.001) trends. The linear increase was significantly higher in the German region compared to the Latin region (95% CIs for the linear trend: 18.04; 21.88 in the German region and 10.86; 14.44 in the Latin region). The positive quadratic trend suggests a higher increase in the German region, and the negative quadratic trend suggests a lower increase in the Latin region (Supplementary Figure S2).

## Discussion

From 1998 to 2020, we found a sharp increase in the case rates of psychiatric hospital admissions for CRD. The majority of cases were found in adolescents and young adults (aged 15–44), with a larger increase in men compared to women.

A general increase in inpatient cases with a psychiatric-related primary diagnosis in Switzerland over the past 20 years is well-documented and known to be multifactorial (e.g., healthcare system immanent determinants, growing sensitization to mental health problems) [16]. In our study, we found a sharp relative increase in case rates for psychiatric inpatient CRD compared to both comparison conditions, both of which either increased more gradually (alcohol-related disorders) or not at all (schizophrenia spectrum disorders). Furthermore, the CRD case frequencies were high predominantly in the 15–24 age group.

The increase in the CRD case rates found in our study corresponds to the findings of a German nationwide investigation of absolute case frequencies in a comparable time period [12]. However, while our study showed an increase in both hospitalization for cannabis dependence and harmful use, the study conducted by Gahr et al. found a much higher increase in hospitalization for cannabis dependence than for harmful use. In relation to cannabis-related psychotic disorder, we found a similar increase to the German cohort of absolute frequencies over the study period. This trend of increased hospitalizations due to CRD was also found in a study cohort in Canada that showed a significant increase in hospitalizations for cannabis-related psychotic disorders [13].

The increase in the demand for inpatient treatment was not correlated with dramatic changes in the prevalence of cannabis use behaviors in the general population in the corresponding time period in Switzerland. As described previously, addiction monitoring in Switzerland showed a slight increase in cannabis use over the last 15 years and even a slight decrease in problematic use from 5.3% in 2004 to 2.8% in 2016 [8].

In contrast to the German cohort, we identified specific subgroups with an elevated risk for hospitalization due to CRD. We found an increase in hospitalizations of cannabis users predominantly in the younger age groups even though the proportion of younger age groups in the Swiss population as a whole slightly decreased [14]. This finding was correlated with a significant increase in the prevalence of monthly cannabis use in the 15–39 age group in Switzerland (women 1.6% in 1992 compared to 3.4% in 2017; men: 4.3% vs. 9.1%) [7]. According to current literature, cannabis use predominantly begins in adolescence and only rarely begins after age 30 [17]. One possible interpretation for the documented increase in CRD hospitalizations in Switzerland may therefore be an increase in the frequency and quantity of cannabis use in young individuals. This behavior has been found to be an important factor underlying the manifestation of severe psychiatric conditions [18, 19]. Further, adolescents have a higher susceptibility to developing mental health problems due to harmful cannabis use, as multiple maturational processes are ongoing in the endocannabinoid system during this critical period [20, 21].

Men were the second risk group in our sample to show a significant trend of increasing hospitalizations because of CRD. This corresponds to evidence that cannabis use disorders (CUD) may longitudinally manifest and develop differently according to sex. For example, men have an earlier age of onset and a higher probability of cannabis use [22]. Men also have a two-fold risk of continuing cannabis use when compared to women and higher risk rates of developing CUD [22, 23]. However, the general finding that men are overrepresented in addiction treatment settings could be biased due to specific facilitators and barriers to women seeking addiction treatment, as has been shown in clinical investigations [24].

In our study, we found increasing case frequencies of inpatient cannabis-related psychotic disorders. Scientific evidence suggesting that cannabis-related psychiatric problems are multicausal and multifactorial (e.g., THC potency, quantity, and frequency of use) and depend less on cannabis use alone [25, 26] has been growing. In this context, cannabis-related psychotic disorders require special attention given their potential to worsen mental health and have lifelong consequences [27]. Although cannabis-related psychotic episodes and long-term schizophrenia often are elusive, growing evidence suggests an interdependence between the two conditions [28]. Recently, a Danish nationwide longitudinal study found an absolute increase in schizophrenia incidence as well as a 3- to 4-fold increase in the proportion of cases of schizophrenia associated with CUD over the past 20 years [29]. The decrease in psychiatric hospitalizations for schizophrenia spectrum disorders found in the current study is inconsistent with the Danish study findings. However, our observational cohort included inpatient cases rather than individuals and thus, general conclusions regarding the prevalence of schizophrenia in Switzerland or causal inferences between cannabis use and psychotic episodes could not be drawn.

Our study has several limitations. First, with the general increase in cannabis use, consumers, physicians, and patients might be more willing to report cannabis use and CRD. Therefore, the increase in CRD in our study may have been affected by reporting bias. Second, the data analyzed in this study represent the number of hospitalizations and not the number of individuals hospitalized; therefore, several individuals might have had two or more hospital stays. Thus, any interpretation of the relative and absolute increase in frequencies reported in our study must consider the possibility of recurrent hospitalizations of severely and chronically affected individuals with CUD. Third, in 2018, the Swiss healthcare system changed its remuneration from a daily rate to a diagnosis-related group flat rate that incentivizes short hospital stays. Consequently, this system increased hospital re-admissions and repetitive hospitalizations of the same individual. However, its effect on our study data may be less relevant since the impact of this new financial system on the duration of hospitalization may not have taken effect yet [30]. Fourth, we were unable to include additional demographic data such as socioeconomic status, ethnicity, or geographic specificities, which limited our understanding of the findings. Sixth, our data did not include information about whether a patient was admitted to the hospital due to herbal or synthetic cannabinoid use. The information on ingestion of other drugs such as cocaine or amphetamines was also not available. Additionally, as we did not include secondary psychiatric diagnoses in our study, CRD hospitalizations might have been underestimated. Seventh, the use of cannabis with higher potencies of THC or synthetic cannabinoids was not captured in the prevalence data. Therefore, we could not assess whether changes in cannabis composition over time correlated with the hospitalization data. Finally, we performed trend analyses using *n* = 23 years, which is a relatively small sample size for a time series analysis.

Our study is the first to analyze CRD inpatient case rates in Switzerland and we found a sharp rise in hospitalizations. There are several implications that can be deduced for the affected population, policymakers and researchers: First, the current Swiss regulatory framework for cannabis, which is mostly non-existent, needs to be reviewed considering the increase in CRD hospitalizations. Future studies should assess whether a different regulatory system for cannabis would alter this worrying trend of increasing psychiatric hospitalizations, in particular for adolescents and young adults. Moreover, the regulation of cannabis may help to protect young people from harmful cannabis use. Finally, more effective and personalized treatment should be made available within the healthcare system as cannabis use continues to evolve in Switzerland.
