# Supplementary material for: A Nationwide Study of Inpatient Case Rate Incidence of Cannabis-Related Diagnoses in Switzerland
Source: Int J Public Health. 2022 Dec 21;67:1605554. doi: 10.3389/ijph.2022.1605554 (PMC9811405; doi:10.3389/ijph.2022.1605554)

Supplementary Material for

A nationwide study of inpatient case rate incidence of cannabis-related diagnoses in Switzerland

Supplementary Table S1……………………………………………………………………..2

Supplementary Table S2……………………………………………………………………..4

Supplementary Figure S1……………………………………………………………………6

Supplementary Figure S2……………………………………………………………………7

Supplementary Table S1. All psychiatric main diagnoses, alcohol-related diagnoses, psychosis-related diagnoses and cannabis-related diagnoses (CRD) with sex, language region, age groups and specific CRDs in the years from 1998 to 2009

	1998	1999	2000	2001	2002	2003	2004	2005	2006	2007	2008	2009
**Swiss Population**	7096465	7164444	7204055	7255653	7313853	7364148	7415102	7459128	7508739	7593494	7701856	7785806
**Psychiatric Main Diagnoses**	50182	60946	71747	75295	80696	82771	83780	81769	127004	131528	144143	85494
**Cannabis-related Main Diagnoses**	185	244	306	355	369	428	461	549	627	464	653	489
**Percentage %**	0.37	0.4	0.42	0.47	0.45	0.51	0.55	0.67	0.49	0.35	0.45	0.57
**CRD case rates**	0.0026	0.0034	0.0042	0.0049	0.0051	0.0058	0.0062	0.0073	0.0083	0.0061	0.0084	0.0063
**Alcohol-related Main Diagnoses**	8501	9953	11490	11853	12529	12493	13230	12718	14273	16604	18568	14605
**Percentage %**	16.94	16.33	16.01	15.7	15.5	15.09	15.79	15.5	11.23	12.62	12.88	17.08
**Psychosis-related Main Diagnoses**	7896	8999	10533	10683	11949	12230	12296	12566	16956	14003	14344	12276
**Percentage %**	15.73	14.7	14.6	14.1	15.3	14.77	14.67	15.36	13.35	11.64	9.95	14.35
**Gender**												
**Female**	58	64	62	92	84	114	92	101	152	94	127	94
**Male**	127	180	244	263	285	314	369	448	475	370	526	395
**Language Region**												
**Latin (French and Italian)**	74	85	161	170	215	232	234	319	389	253	414	233
**German**	111	159	145	185	154	196	227	230	238	211	239	256
**Age Groups**												

**Age 15-24**	95	106	162	209	195	231	234	345	294	228	248	255
**Age 25-44**	80	103	130	115	144	162	191	155	257	196	360	200
**Age 45-64**	4	18	10	16	22	25	31	36	66	29	43	23
**Age 65+**	2	9	2	1	1	4	4	6	1	5	6	1
**CRD Percentage % 25-44**	1.1	1.2	1.3	1.2	1.3	1.5	1.7	1.4	1.3	1.5	2.5	1.8
**CRD Percentage % 15-24**	7.9	7.1	8.6	9.2	7.9	8.9	8.6	12.2	5.6	6.9	8.2	10.4
**Cannabis-related specific Diagnosis**												
**F12.0**	27	39	35	59	44	68	45	53	41	23	45	30
**F12.1**	14	41	51	67	49	54	76	74	88	63	71	82
**F12.2**	80	73	108	126	154	155	225	257	287	222	386	246
**F12.3**	4	1	0	4	1	2	7	4	4	6	6	6
**F12.X***	8	10	15	15	19	22	12	13	21	9	17	10
**F12.5**	52	80	97	86	96	125	99	146	180	141	115	117

X* comprises the following CRDs according to ICD-10 : F12.4, F12.6, F12.7, F12.8, F12.9

Supplementary Table S2. All psychiatric main diagnoses, alcohol-related diagnoses, psychosis-related diagnoses and cannabis-related diagnoses (CRD) with sex, language region, age groups and specific CRDs in the years from 2010 to 2020

	2010	2011	2012	2013	2014	2015	2016	2017	2018	2019	2020
**Swiss Population**	7870134	7954662	8039060	8139631	8237666	8327126	8419550	8484130	8544527	8606033	8670300
**Psychiatric Main Diagnoses**	89806	97877	90888	92742	95306	97877	101385	104563	103131	105025	103914
**Cannabis-related Main Diagnoses**	629	662	673	815	854	858	824	864	860	854	961
**Percentage %**	0.7	0.67	0.74	0.87	0.89	0.87	0.81	0.83	0.83	0.81	0.92
**CRD case rates**	0.0079	0.0083	0.0084	0.01	0.01	0.01	0.0098	0.01	0.01	0.0099	0.11
**Alcohol-related Main Diagnoses**	16126	16369	15530	15595	15988	15626	15703	15868	15784	16755	16582
**Percentage %**	17.09	16.72	17.08	16.81	16.77	15.96	12.88	12.62	12.37	12.3	15.95
**Psychosis-related Main Diagnoses**	12590	12309	11549	11917	12272	12686	13068	13204	12763	12924	13241
**Percentage %**	14.01	12.57	12.71	12.84	12.87	12.96	12.89	12.66	12.37	12.3	12.7
**Gender**											
**Female**	129	149	124	181	188	192	187	161	171	178	218
**Male**	500	513	549	634	666	666	637	703	689	676	743
**Language Region**											
**Latin (French and Italian)**	333	332	339	404	400	392	353	394	347	351	406
**German**	296	330	334	411	454	466	471	470	513	503	555
**Age Groups**											
**Age 15-24**	349	317	355	375	394	443	391	397	411	423	465

**Age 25-44**	233	282	264	364	379	345	368	387	373	358	425
**Age 45-64**	37	56	45	64	78	56	59	68	70	61	58
**Age 65+**	5	6	1	5	4	3	4	1	6	6	4
**CRD Percentage % 25-44**	2	2.4	2.5	3.3	3.4	3.1	3.3	3.4	3.4	3.2	3.6
**CRD Percentage % 15-24**	13.3	12.2	15.3	16.71	16.5	19.7	17.3	16.7	16.5	16.1	17.1
**Cannabis-related specific Diagnosis**											
**F12.0**	42	34	37	49	48	57	34	41	45	60	69
**F12.1**	95	90	92	119	125	117	110	109	96	69	67
**F12.2**	339	371	376	480	481	490	494	489	482	457	485
**F12.3**	10	4	1	10	7	14	6	8	11	15	20
**F12.X***	13	18	14	19	21	25	16	16	18	26	24
**F12.5**	139	139	127	134	173	161	159	194	199	197	232

X* comprises the following CRDs according to ICD-10 : F12.4, F12.6, F12.7, F12.8, F12.9

Supplementary Figure S1. Annual frequencies and estimated (linear) trend line for inpatient cases with cannabis related diagnoses (CRD). CRD-R includes other CRD Diagnoses

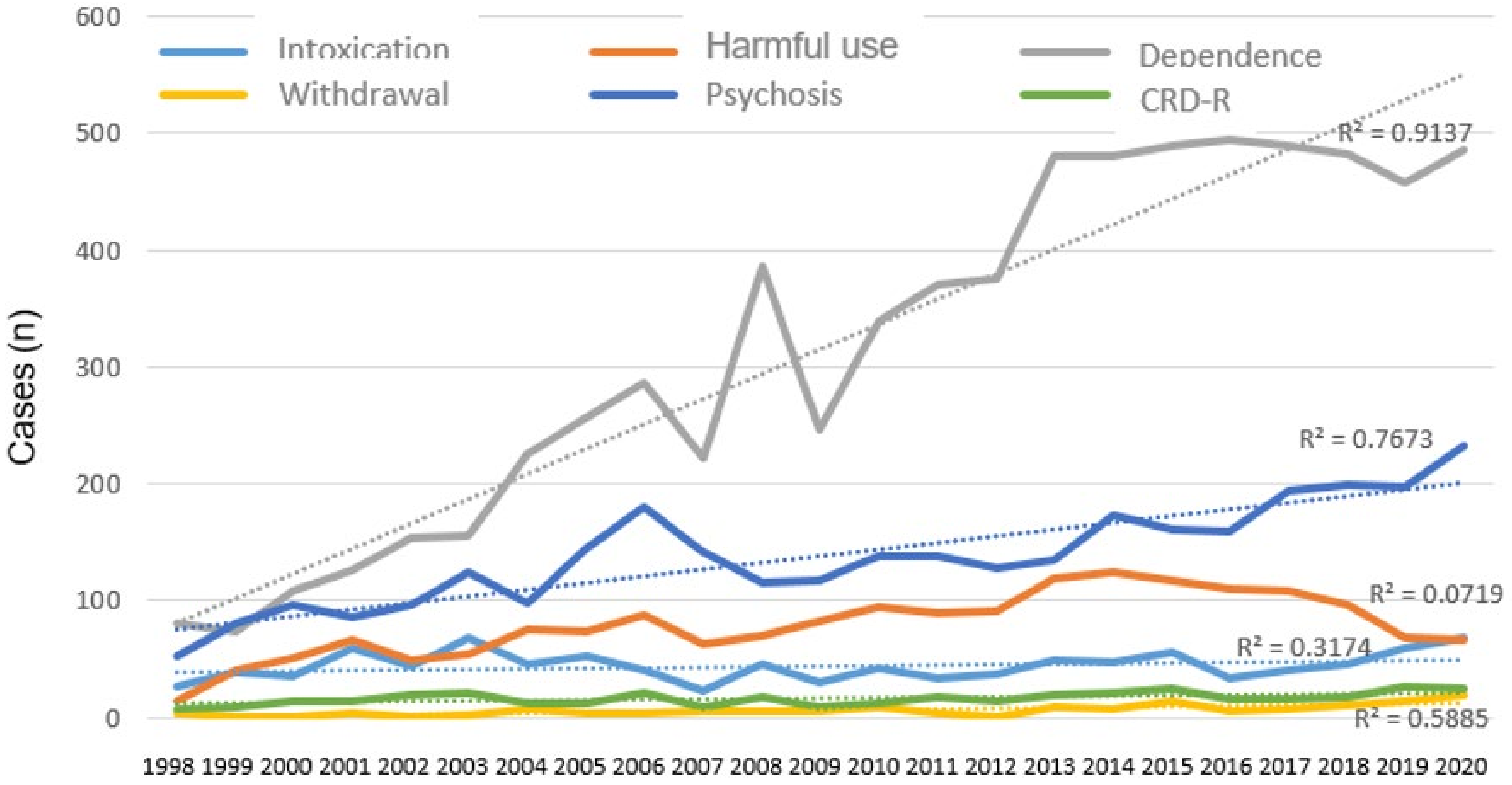

Supplementary Figure S2. Annual frequencies for inpatient cases with cannabis-related diagnoses (CRD) in Latin and German language regions

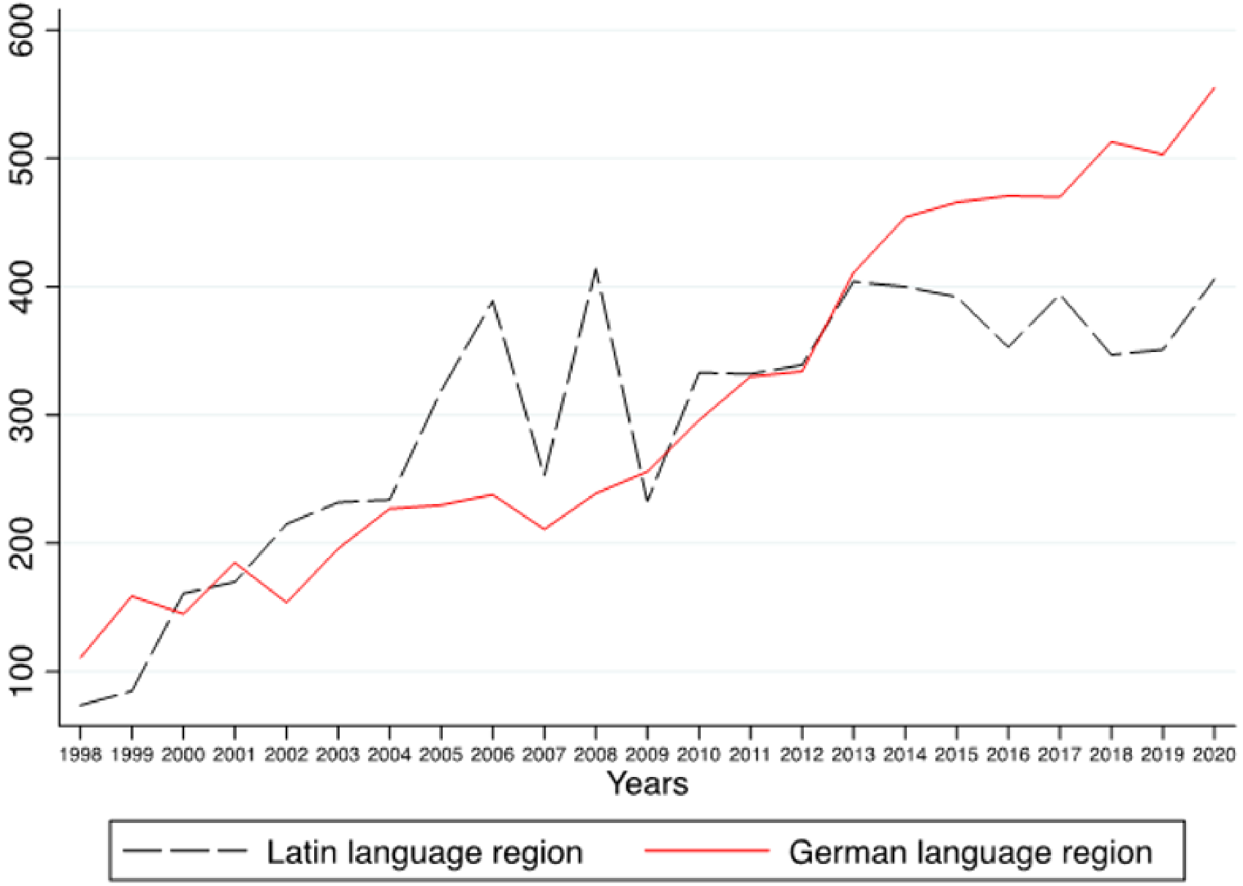

Supplement: Supplementary file 1 [file DataSheet1.PDF]
